# A systematic review: Children & Adolescents as simulated patients in health professional education

**DOI:** 10.1186/s41077-015-0003-9

**Published:** 2016-01-11

**Authors:** Andrée Gamble, Margaret Bearman, Debra Nestel

**Affiliations:** 1 Health Science & Biotechnology Department, Holmesglen Institute, Chadstone, Victoria Australia; 2 HealthPEER - Health Professions Education and Educational Research, Monash University, Melbourne, Victoria Australia; 3 Simulation Education in Healthcare, School of Rural Health, HealthPEER, Faculty of Medicine, Nursing and Health Sciences, Monash University, Melbourne, Victoria Australia; 4 Graduate Programs in Surgical Education, University of Melbourne, Parkville, Victoria Australia

**Keywords:** Simulation, Education, Simulated/standardized patient, Child, Adolescent, Nursing, Health professionals, Systematic review

## Abstract

Simulated patients (SP) contribute to health professional education for communication, clinical skills teaching, and assessment. Although a significant body of literature exists on the involvement of adult SPs, limited research has been conducted on the contribution of children and adolescents. This systematic review, using narrative summary with thematic synthesis, aims to report findings related to children/adolescents as simulated patients in health professions education (undergraduate or post-graduate). A systematic review of qualitative and quantitative literature published between 1980 and September 2014 was undertaken using databases including CINAHL, Ovid Medline and Scopus. The lack of literature related to the employment of children and adolescents in nursing education dictated the expansion of the search to the wider health professions. Key search terms related to the employment of children and adolescents in health professional education programs. A total of 58 studies reduced to 36 following exclusion based on abstract review. Twenty-two studies reached full text review; following application of inclusion and exclusion criteria, 15 English language studies involving children and/or adolescents in simulation formed part of this systematic review. Five key themes emerged: Process related to recruitment, duration and content of training programs, support and debriefing practice, ethical considerations, and effects of participation for key stakeholders such as children and adolescents, parent and faculty, and learner outcomes. The results suggest that the involvement of children and adolescents in simulation for education and assessment purposes is valuable and feasible. The review identified the potential for harm to children/adolescents; however, rigorous selection, training and support strategies can mitigate negative outcomes. The ability of children to portray a role consistently across assessments, and deliver constructive feedback remains ambiguous.

## Background

Learning through clinical practice has traditionally been the mainstay of practice-based health professional education programs. As an example, nursing education has reducing access to clinical placements and exposure to appropriate clinical learning environments is less certain. The inclusion of clinical practicum into the first year of many nursing undergraduate education programs, both nationally and internationally, has also necessitated students are exposed to professional, psychomotor and developmentally appropriate communication skills earlier than was historically necessary. Holistic, realistic and safe approaches to learning professional and psychomotor skills prior to patient exposure are necessary. These approaches need to equip the student with useful and transferable skills they can apply in the complex clinical environment [1]. This is especially critical now that the National Council of State Boards of Nursing (NCSBN) study has identified the potential for replacing at least some proportion of clinical hours with simulation [2]. Results from this study of nursing students who had a proportion of their clinical hours replaced with simulation (10 %, 25 % or 50 %) indicates no statistically significant differences between the groups at the end of their nursing program in relation to clinical competency, comprehensive nursing knowledge assessments, and NCLEX pass rates. Six months into employment, no statistically significant differences were identified by their managers in relation to clinical competence or readiness for practice.

Paediatrics is a specialised field; the nuances and specific characteristics of children and adolescents must underpin all education approaches. However, learners with limited personal experience or exposure to children can find communication, interaction, assessment and the provision of developmentally appropriate care difficult [3]. Without the opportunity to apply, practise and evaluate these important skills prior to clinical placement, learners may find assimilation into the paediatric clinical environment difficult [1]. Simulation has been identified as a powerful active learning strategy, and an important part of health professional education. Learners are immersed in realistic situations where they have an opportunity to engage in skills-based scenarios in a ‘patient-safe’ environment. Simulation can offer learners exposure to professional domains such as teamwork, communication and time management and provide participants with almost all the essential components of a real situation. This exposure can then serve as a reference point to guide actions should the situation arise during clinical exposure [4].

Simulated patients (SPs) are defined as well people who have been trained to portray patients with a specific condition in a realistic way [5]. Adult SPs have contributed to clinical skills teaching since proposed by Barrows and Abrahamson in the late 1960s [6]. The benefits of SPs are numerous and widely researched in literature. Often employed in healthcare education programs to portray roles, SPs enable students to immerse more fully in the reality of a clinical situation [7, 8]. Working with SPs also reduces the haphazard nature of student/patient encounters in the clinical environment, resulting in standardization and fairness in exposure to learning opportunities and therefore also during assessment [9]. SPs can also communicate, interact and provide the learner with humanistic and developmentally appropriate responses that are difficult to replicate in a manikin-based program.

There is an obvious threat to patient safety with novice health professionals practising skills on real patients. By contrast, SPs are usually more widely accessible, able to portray a role multiple times and can work in situations where a real patient would be inappropriate. Consistent and standardized role portrayal also makes SPs suitable for clinical assessments where neither manikins nor real patients would be appropriate [10].

Paediatric education is most commonly introduced using a range of technologically diverse manikins. Although an excellent medium for teaching and learning in some areas, manikins can limit realism as their communication and behaviour is often unrealistic and may not reflect the developmental stage required by the simulation role [11]. In addition, manikins are unable to replicate the well child, or the child with a normal childhood illness, both of which are critical to adequate clinical preparation for practice. For these reasons, true learner engagement and immersion is often difficult to achieve.

Communication with children and adolescents is an essential element of health professional socialization, but is difficult to teach and/or assess in the educational setting. Clinical environments are often identified as a more appropriate setting for this learning to occur. However, a reducing number of placements, disparity in the quality and available learning experiences during placement and parental control over access to sick children has made learning more challenging [2]. An ever present patient safety agenda and ethical concerns associated with utilizing sick children for learning can also impact on student exposure to learning opportunities.

It is reasonable to suggest that children and adolescents should become a more important part of SP methodology. Simulation based education with children/adolescents has been used for many years to successfully engage students in various domains of learning. Across the physical examination and professional skills continuum, studies have demonstrated the value of adolescent SPs to education and assessment programs, particularly those related to communication [11].

In paediatrics, the use of children as employed SPs has long been questioned with regard to ethics and the examination of validity, reliability, and feasibility [12]. There are also inherent difficulties in employing real children. Ensuring children are adequately prepared, trained and supported using developmentally appropriate strategies can be challenging. The ethical considerations, particularly of employing children below the age of consent, must be considered when working with children and/or adolescents. Additionally, the age of the child, the role they play and the duration of engagement are crucial considerations.

### **Definition of** t**erms**

Child SPs (CSPs), for the purpose of this review, are aged between 5–12 years, while adolescent (ASPs) refers to participants aged 13–19 years. We use the Child and Adolescent SPs (CASPs) to include babies, CSPs and ASPs. In choosing to differentiate between children and adolescents, a developmental approach was considered appropriate due to the ambiguous nature of consent and Victorian (Australia) labour laws regarding employment of children. This review considers the employment of children for simulation as work in the entertainment industry; as such, all ages from infancy through to adolescent can be employed for a variable duration dependent on age and employment guidelines [13].

### Aim

The aim of this systematic review is to analyse the available literature and generate discussion and recommendations for future research related to the involvement of CASPs in health professional education.

### Review question

This review aims to answer the following question: What is reported in the literature regarding children and adolescents who work as SPs in health professional education?

## Methods

A systematic search was undertaken for qualitative, quantitative and mixed method papers related to employment of CASPs in all health professional education programs.

### Search strategy

Between June – September 2014, seven databases were searched (CINAHL, Ovid Medline, PsychInfo, Google Scholar, Scopus, Cochrane database of systematic reviews, and Informit,). Reference lists from all papers and grey literature were also searched. The search solely focused on literature written in English; no date restrictions were applied. Search terms fell into three broad categories: Education, simulation and developmental stage, (Table 1). For example, a CINAHL search was conducted using the terms; simulated patient AND adolescent OR child AND education.

**Table 1:** Key Search Terms

Education	Simulation	Developmental Stage
Nursing	Simulated patient	Child
Nurs*	Simulation	Children
Medicine	Sim*	Adolescent
Health professions	Standardized patient	Paediatric
Medical students	SP	Toddler
Undergraduate		Pre-School*
Postgraduate		School Age
Education		Teen*
Role play		Teenager
Communication		
Patient simulation		
Scenario		
Nurse education

**Table 2:** Inclusion/Exclusion criteria

Inclusion Criteria	Exclusion Criteria
All studies focusing on children/adolescents as SPs	Content focused on adult SP rather than child/adolescent SP
	SPs not children or adolescents
Health education program	Non-health education program
Peer reviewed	Not peer reviewed
All study designs including reviews	Research or review not focused on topic
English	Manikin based programs

### Study selection

Initially, the involvement of children and/or adolescents as SPs in nursing education programs was the intended primary focus. However, limited numbers of papers dictated expansion to all health professional groups, undergraduate and post-graduate students and professional development programs. This review considers multiple research methods, including randomized control trials, control trials, qualitative studies, observation and exploratory studies. Studies written in English with reference to children and/or adolescents as SPs were included without application of date restriction. All peer-reviewed studies, including literature and systematic reviews, were included.

### Data extraction

More than 1000 studies were identified in the initial search. This number was reduced to 60 through application of the exclusion criteria to the title alone. A large majority of the literature was further excluded based on review of title and abstract, resulting in 22 full text studies Table 2. Full review of these 22 studies resulted in extraction of data from the final fifteen papers presented in Table 3.

**Table 3:** Data Extraction Table

Reference	Study location	Sample	Study Purpose	Study design	SP Population	SP preparation	Outcome Measures	Learner Outcomes	SP Outcomes	SP related Considerations
**Austin** ***et al.*** [17]	**USA**	Nursing students *N* = 263	Identify the Impact on health professionals & children following their involvement in disaster preparedness simulation	Qualitative evaluation	16 children 6–15 years	Multiple sessions targeting different areas of preparation & role practice	Parental interview to gain understanding of child & parent experiences; Written evaluations from nursing students about nursing process, confidence & knowledge gain	Identified 3 main nursing roles during mass casualty; assessment, triage & interventions; work in multi-professional team to improve rapid assessment & decision making skills; improved confidence (52 % reported some confidence, 21 % very confident & 19 % slightly more confident); 42 % gained awareness of hectic nature of mass casualty	Parents reported children had an increased awareness of disaster-readiness; Children loved the experience; Parents felt education & preparation was excellent; Would allow child to participate again	Parental consent & presence; School support; Nurse dedicated to 1:1 support during Sim; Avoidance of critical events; ‘Opt out’ option
**Blake** ***et al.*** [22]	Canada	Final year medical students *N* = 57 intervention group *N* = 35 in control group	To determine if feedback from adolescent and mother leads to improvements in 4th year medical students’ psychosocial interviewing To evaluate whether this skill persists in the long term (2–12 months post intervention, average 6.6 months)	Prospective randomized double blind study with 3 arms; Intervention group received feedback from adolescent SP & SP mother after 2 interviews, 4 weeks apart. 2nd intervention group received feedback once after 2nd interview only. 3rd group did not participate in interview	9 SPs as mothers 10 female adolescent SPs	Standardized feedback training Adolescents guided by SP mothers to give feedback Adolescent focus group	Pre-test review by psychologist using modified Calgary-Cambridge guide of interview with adolescent and mother SP Post-test review of second interview 4 weeks after pre-test Evaluation of knowledge & psychosocial interviewing scores on 2 OSCE stations	Group who received feedback after 1st interview scored better on post-test; Both intervention groups had higher scores in psychosocial inquiry station in OSCE but not in knowledge; Adolescent interviewing skills can be taught & retained up to a year.		Time spent recruiting & training is important.
**Blake** ***et al.*** [23]	Canada	*N* = 54 final year medical students	To identify any adverse effects on adolescents who regularly undertake risk-taking roles; to capture the viewpoint of adolescents over time; to describe the training and monitoring process for adolescents as risk-taking SPs	Prospective study involving control groups	*n* = 11 female adolescents aged 13–15 Y Control *n* = 6 SPs of same age & grades completed	Information session	SP:Pre & post Interviews using Achenbach’s youth self-report & Piers Harris Children’s self-concept scale; Focus groups; Parental interview & questionnaire		PRE: SCS &YSR not in clinical range of concern for study or control groups; Focus groups: Develop attachment to SP mother; Wish to come out of character to give feedback; Benefitted from experience but SP work did lose glamour and become a job Parent interview: Saw as opportunity for adolescent empowerment & to better understand how difficult it is for doctors, no increased interest in risk-taking behaviours	Recruitment & screening important; Debrief; Exit strategy; Paid
**Bokken** ***et al.*** [29]	Netherlands	2nd year medical students over 5 years	Evaluate the views of teachers, students & adolescent SPs regarding the SP program; Evaluate the extent to which all 3 felt the program had changed over 5 years; Evaluate the lessons learner 5 year experience of the SP program	Pre/post tst	*n* = 16 adolescent girls 13-19y *n* = 2 males	Introduction session & feedback training	Students rated quality of SP role performance & feedback using Maastricht assessment of simulated patients (MaSP); Adolescent SP questionnaire about their experience; Faculty completed questionnaire about SP consultation, quality of feedback & role play & students reactions	Authenticity of encounter 7.5-8/10, adolescent SP fits role & stays in it; general performance of adolescent SP decreased over 5 years; Faculty saw encounter as authentic, able to address specific aspects of communication not able to be assessed in other ways, SPs able to give natural & spontaneous feedback	No personal disadvantage; Some difficulty with feedback; 8 role plays per day ideal; No differences in evaluation across 5 years	Parents advised by adolescent; Paid; Individualized role
**Bokken** ***et al.*** [30]	Netherlands	Medical students *N* = 341	Evaluation of effects on adolescent SP of performing a role, the quality of their role playing and feedback	Descriptive evaluation	Adolescents aged 16–18 *N* = 12	Role developed with adolescents based on their own experience. Role related & feedback training	Students rated quality of SP role performance & feedback using MaSP; Adolescent questionnaire about effects of SP role; Faculty evaluation of, quality of feedback & role play	Learners indicated satisfaction with quality of role play & feedback; Student doctor & observer rated SP performance differently; Teachers noted a positive & authentic experience & acknowledged students may feel attracted to SP	Positive experience; Easier playing a role close to own experience; Need more feedback training	Given letter for parents but not mandatory to give it to them; Paid for their time
**Brown** ***et al.*** [18]	USA	Medical students & Residents	Description of a pilot program to aid in training residents & medical students in complex interviewing skills addressing adolescent mental health issues	Qualitative	Children & adolescents aged 9–19 years	2 training sessions Not involved in case preparation	Resident & medical student questionnaire about the program & achievement of learning outcomes, Focus groups with child–parent SP dyads focused on preparation for roles, reactions to participation, ability to give feedback, reactions to roleplaying with biological/SP mother	Learning outcomes achieved & mostly positive program feedback – 2 learners preferred SP approach whilst 3 preferred lecture format	Child: Fun; empowering; contribute to learning for doctors; financial benefit SP & SP parent: Training was good preparation; Mixed reaction to providing feedback – some would prefer to give to faculty instead of directly to learner ; Varied opinion about biological/SP mother	No psychological follow up Children made links with personal experiences Don’t need own parent present ‘Opt out’ clause Paid
**Feddock** ***et al.*** [19]	**USA**	Medical students *N* = 95 intervention *N* = 91 control group	Determine effect of adolescent medicine workshop on knowledge & clinical skills	Randomised controlled trial Intervention: Medical students participating in adolescent medical workshop Control: Medical students in alternative workshop			End of year clerkship exam with adolescent SP encounters; 3rd year clinical exam; written exercise & questions specific to adolescent medicine on clerkship written exam	Performance of intervention group higher on clinical skills & written exam		
**Hanson** ***et al.*** [24]	**Canada**	2nd year medical students	Evaluation of adolescent selection methods & simulation effects for low & high stress roles in a psychiatry OSCE	Randomised controlled trial SP assigned to low stress/high stress role or control group	Secondary school age adolescents	Information & training session Employment & psychological screening	Simulation impact questionnaire; Interview; Focus group; Adolescent self-perception profile; Achenbach behaviour questionnaires; Parental version of simulation impact questionnaire; 3 months after participation – interview; project role questionnaire to identify comfort enacting various roles		Identify good/bad doctors; Importance of training for SP work; Some adverse effects on relationships with peers, parents & school performance; No pre/post change in self-perception or Achenbach questionnaire; Discomfort with sexually explicit questions Parents reported no adverse effects, small increase in self-confidence, job skills & sense of responsibility	Adolescent & parent consent
**Hanson** ***et al.*** [25]	Canada		Evaluating safety of suicidality sim	Pre-post	*N* = 24 14–17 years	Information session Screening Group training	Suicidal ideation questionnaire; Reynolds adolescent depression scale; behavioural measures		No deterioration in mental health status; Suicidality role showed negative reaction with; 2 reports of brief depression	Consent Ethics approval MH specialist Stress relief methods debriefing
**Hanson** ***et al.*** [26]	Canada	*N* = 34 paediatric residents	Determine association between simulation discomfort & mental illness stigma	Randomised controlled trial	*N* = 24 14–17 years Randomised to suicide/depression or cough scenario	4 hours training & rehearsal	Project role questionnaire		Discomfort with sex questions due to lack of knowledge; Adolescents experienced in mental illness roles anticipated greater comfort portraying subsequent stigma associated roles	Consent Ethics approval
**Lindsey-Lane** ***et al.*** [20]	USA	Paediatric medical residents *N* = 56	Obtain qualitative data about the appropriateness, feasibility & responses of child SPs in CSA	Observational	*n* = 11 aged 7–16 *n* = 9 adults paired with children	Training sessions until consistency gained between history, PE & professional skills	Adult SP: Patient encounter checklists; Child SP gave overall patient satisfaction rating on checklist; SP focus groups with child/adolescents or SP and real parents; Residents completed questionnaires related to realism & challenge	Residents ratings low for fairness (2.9/5), but higher for enjoyment (3.1), realism (3.9) & challenge (4.1)	Child & adult SP satisfaction ratings concordant; Parent Focus Groups gave positive feedback about learning, working hard at a real job; SP parents noted child SP had negative reactions if ignored or talked down to Children found experience at times exciting, nerve wracking & boring, tiring by the end of 6 hours, but good to earn money	Careful selection, in-depth training and debriefing by individuals experienced in communication with children
**Pullon** ***et al.*** [27]	NZ	*N* = 69 medical students	Assess consultation skills teaching & risk of harm to involved adolescent SPs	Retrospective evaluation	Adolescent girls (14–18) *n* = 4 *n* = 3 adult SPs	Discussion about suitability of case Training	Student self-evaluation, video tape review of consultations by tutor; Interviews with adolescent SPs; Retrospective student evaluation via focus group	Increased confidence in consultation skills, however no clear effect on clinical performance	Adolescents positive about role, no negative effects but able to identify possible harm if supports not put in place	Parental & student consent Clear criteria of concern
**Rowe** ***et al.*** [28]	Africa	5 rural community & one city health service	To evaluate health care worker performance during consultations	Evaluation survey	6 children aged 6 m-59 m 5 SP mothers	SP mothers: 3 training days 3 months prior and a 2 day refresher just prior to study. No child SP preparation identified	Survey result analysis – client survey & conspicuous observation		No serious problems for SPs	Ethics approval obtained
**Tsai** [12]	**Taiwan**	19 studies – English, searched via Medline	Review use of child SPs & difficulties in using children in assessment of competence	Systematic review	Children as SPs in clinical assessments				Children from infancy to adolescence can participate as SPs in clinical assessments; Children should have a substitute; Can provide feedback; More negative impacts for younger children; Use of children should be avoided for ethical reasons	Only work with children for assessments that cannot be measured by other methods
**Woodward, & Gliva-McConvey,** [21]	USA		Identifying the effects of simulation on children	Qualitative retrospective	*N* = 7 Children 6-18	Random selection from existing pool of child SPs	Focus group		Important skills & information gained; Positive & negative outcomes for younger children; fun can disassociate from role; Mainly positive for older children; Help adults learn; Identify good & bad doctors	Mothers included if children <13 Role close to the child’s personality & developmental age. Greater risk in younger children. Methods to monitor effects on children

**Fig. 1:**
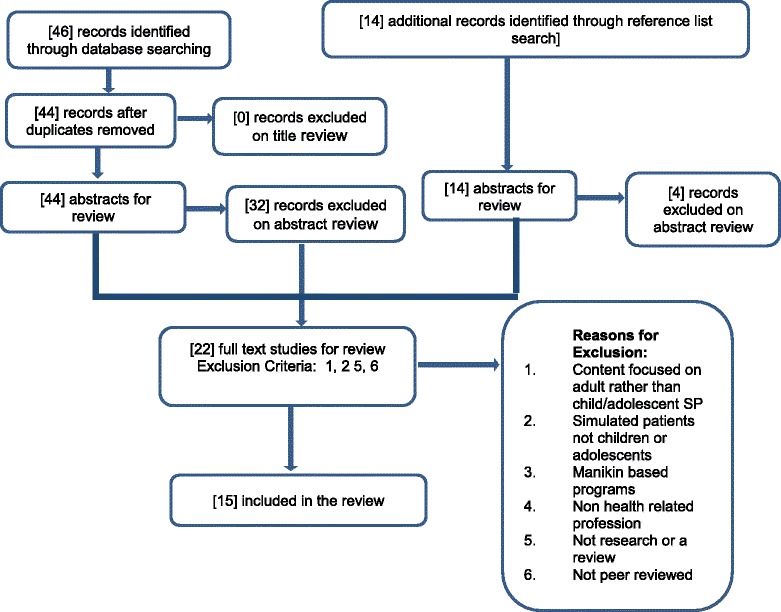
Study inclusion process

The PRISMA diagram has been utilised to represent the study inclusion and exclusion process underpinning this review (Fig. 1). PRISMA is an evidence-based set of terms used for reporting in systematic reviews [14]. Initial exclusion of studies occurred on abstract review; the lack of involvement of children and/or adolescents as SPs, and a primary focus on subjects not directly related to the review topic resulted in the greatest proportion of exclusions at this stage. Twenty-two studies progressed to full-text inclusion with 7 discarded following review. Four main reasons underpinned their exclusion; children/adolescents who although identified in the studies, were secondary in focus to adult SPs; lack of direct correlation between study content and research focus of this review; no explicit identification of research or review methodology and publication of study in a non-peer reviewed journal.

### Assessment of methodological quality

Studies underwent a quality analysis process relevant to their research methodology. Qualitative studies were analysed according to the Criteria for appraising qualitative research designed by Walsh and Downe [15]. This is an 8 item checklist, structured into three sections: stages, essential criteria and specific prompts which further delineate into sub-sections focusing on various criteria related to analysis of qualitative research studies. Quantitative literature was appraised using the Medical Education Research Study Quality Instrument (MERSQI). The MERSQI is a ten-item instrument designed to assess the methodological quality of experimental, quasi-experimental, and observational medical education research studies. The ten items reflect six domains of study quality: study design, sampling, data type (subjective or objective), validity of assessments, data analysis and outcomes [16]. Refer to Table 4 and Table 5 for study assessments.

**Table 4:** Quality Analysis (Walsh & Downe, [15])

Author	Clear statement of purpose	Method consistent with research intent	Sampling strategy appropriate	Appropriate analytic approach	Interpretation	Data used to support interpretation	Researcher reflexivity demonstrated	Sensitivity to ethical concerns	Relevance & transferability
Austin *et al.* [17]	1	2	3	2	1	2	2	1	1
Bokken *et al.* [29]	1	2	2	2	1	1	2	2	1
Bokken *et al.* [30]	1	2	1	2	1	2	2	2	1
Brown *et al.* [18]	1	2	1	2	2	3	2	2	1
Lindsey-Lane *et al.* [20]	1	2	1	2	2	2	4	4	2
Pullon *et al.* [27]	1	1	1	2	1	1	1	1	1
Rowe *et al.* [28]	1	1	2	2	1	2	1	1	1
Tsai [12]	1	1	1	1	1	2	NA	NA	1
Woodward & Gliva-McConvey [21]	1	1	2	1	1	1	3	2	1

Key: 1 = Yes, 2 = Partially, 3 = No, 4 = Unknown

**Table 5:** Quality Analysis (MERSQI)

	Study Design	Sampling	Type of data	Validity of evaluation instrument	Data Analysis	Outcomes
Blake *et al.* [22]	3	2	3	2	2	1.5
Blake *et al.* [23]	2	2	3	3	3	3
Feddock *et al.* [19]	3	2	3	1	2	1.5
Hanson *et al.* [24]	2	0.5	1	1	3	3
Hanson *et al.* [25]	1.5	2	1	2	2	3
Hanson *et al.* [26]	3	2	1	2	2	3

## Results

### Description of Studies

There were 15 included studies; Tables 3, 4, 5 outline overviews of content and quality. Of these studies, 5 were conducted in the USA [17–21] or Canada [22–26] while one study was a systematic review from multiple countries [12]. The remaining four studies originated in New Zealand [27], Africa [28] and The Netherlands [29, 30].

Eleven studies identified the health professional group to which the learner belonged. Nursing students [17] were involved in one study, while 10 studies focused on medical students or physicians. Participant numbers ranged from 34 paediatric residents [26] to 341 medical students [30]. Two randomised control trials were included, and in both cases, baseline data of participants was comparable [19, 23].

In relation to the central focus of the intervention, two studies focused on Objective Structured Clinical Examination (OSCE) or Clinical skills assessment (CSA), eleven related to simulation, while two studies addressed these in combination. Communication was the primary learning outcome for participants in ten studies and four studies related to a combination of communication and physical examination skills.

All studies discussed the experience of children or adolescents as SPs with varying degrees of focus on participant numbers, gender, ages and recruitment strategies. SP participant numbers ranged from four to twenty-four. Ten studies either did not specifically identify gender of the children or adolescent SPs or employed both males and females [12, 17–21, 24–26, 28]*,* three studies focused solely on females [22, 23, 27] and two studies included a small number of males through convenience rather than planning [29, 30]. SPs were recruited from an existing database, following contact with a local community theatre or drama group or from faculty willing to involve their own children. Seven studies focused solely on adolescents whilst five expanded their CASP involvement to children aged 6–7 years [17, 18, 20, 21, 28].

For qualitative studies, the experience of children and adolescents was captured in post simulation interviews and focus groups. Perspectives of CASPs, biological parents and SP parents were sought at variable points after CASP involvement although Austin [17] chose to focus solely on evaluation data collected from parents. In contrast, a selection of studies used a multi-layered approach to analyse effects of participation on adolescent SPs. Tools employed to gather data included pre and post administration of behavioural type questionnaires and specific project surveys designed to assess the impact of role playing on CASP participants [23–26].

In two studies, CASP evaluated the performance of students. Feddock *et al.* [19] provided SPs with case-specific checklists designed to assess adolescent medicine knowledge and general interviewing/counselling skills. While not completing a specific checklist, Lindsey-Lane *et al.* [20] allowed children as young as 7 years to give an overall satisfaction rating on the simulated encounter. Students were also involved in direct assessment of CASP performance. Bokken *et al.* [29] applied the Maastricht assessment of SP (MaSP) to evaluate role performance and quality of feedback provided by adolescent SPs.

The type of outcome measures and associated data collection tools varied widely. A variety of data was captured through the use of questionnaires, interviews, focus groups, assessment results and validated screening instruments. Of note was the repeated focus on the specific outcomes for the child and their ability to give feedback. However, even within these diverse data collection methods, the impetus for many studies appeared to be the identification of risk or adverse outcomes for the child or adolescent.

Whilst diversity in outcome is apparent, most studies chose to refine their focus to specific aspects of learning, most prominent being the choice between clinical skills or knowledge. Limited studies chose to evaluate both of these domains despite their obvious need to inter-link in clinical practice. When both domains were assessed in end of clerkship written and clinical exams, a higher score was attained by those learners receiving SP based education in comparison to those who did not.

In most cases, SP views were included in data collection in those situations where an adolescent rather than a child had fulfilled the SP role. Additionally, those studies that did involve younger children chose to focus more on the evaluation provided by either the child’s biological parent, or the adult role playing their parent within the simulation activity. Perhaps an opportunity exists in this situation for the incorporation of developmentally appropriate evaluation tools as a means to ensure the valuable feedback of children is not omitted.

Longitudinal application and retention of knowledge were not common outcome measures, despite the potential for these to reinforce the value of child and adolescent SPs to educational outcomes. Two studies included these measurements with variation in the result apparent. Although one study indicated the retention of knowledge for up to one year [22], a second paper provided contrast by identifying that even in the short term there was no appreciable positive impact on clinical performance [27].

### Synthesis

All studies were read and reread numerous times to obtain an overall sense of the data. Content that stood out as meaningful was identified and utilised as the basis for theme formation. Studies were initially analysed by the primary researcher, with some further checking for themes undertaken by secondary authors [31].

Analysis of the 15 studies identified five critical considerations that may impact on the inclusion of children and/or adolescents in simulation based education or assessment programs. These are: recruitment, training, participation and support, ethical issues and the impact of CASP involvement on the learner. Two key additional themes emerged from the analysis: parental and child perspectives.

### Critical considerations

#### Recruitment

The recruitment and screening processes for children and adolescents are clearly important [20–24, 27]. Ensuring adolescents are able to cope with the simulation content, are mature and have a sense of reality about the role, particularly if it involves risk-taking, is vital. Careful selection appears to correlate with more successful and realistic role portrayal [23] as does matching developmental age and personality with the content and expectations of the role [12, 21].

Studies that identify source of recruitment indicate that local schools and community theatre groups in close proximity to the simulation location, and employing children of faculty and their friends were the most effective in finding suitable participants [17, 18, 20, 21, 22, 24, 27, 29, 30]. Collaborating with schools is considered important during selection processes. Teachers are ideally situated to identify suitable students, such as those who have interest in drama, or conversely, those who cannot afford to miss time from school [18, 23].

Recruitment processes ranged from a convenience sampling approach to implementation of strict pre-selection testing using a various assessment tools. Brown *et al.* [18] only employed children with no personal experience of the condition they were to simulate. In contrast, Hanson *et al.* [23–25] and Blake *et al.* [23] used a more rigid approach to selection with a combination of validated tools investigating constructs such as suicidal ideation and adolescent depression [25].

#### CASP training

Detail about preparatory training time and content was difficult to gauge. The number of hours in training, if specified, ranged between 2, 4 and 8 hours [20, 23, 25]. The content of training also varied within the studies with options encompassing the core role of an SP, specific case training [20, 25], tips to remember the role and multiple practice opportunities. In studies related to adolescent mental health or risk-taking behaviours, the need to engage specialists in training was acknowledged [25, 26].

The duration, level and content of preparatory programs appears to be depend on the age of the child and the role content. Where there is critical content and CASPs are involved in delivery of feedback, their preparation is more time intensive and detailed. In contrast, studies where feedback is not given directly by the child, or the content is less psychologically stressful, the time dedicated to training decreases.

The ability to give effective feedback to participants was identified as a key component of the CASP role [18, 22, 29, 30]. However, this was not always recognized or acted on during training. Studies indicate that CASP feedback is powerful, but there are mixed reactions from both children and adolescents [18, 29, 30]. Brown *et al.* [18] identified that whilst one adolescent felt uncomfortable giving direct feedback to a student, another reported that the protective mantle of the role and the perceived importance of the information enabled them to feel more comfortable.

Bokken *et al.* [30] suggest that a role developed in consultation with, and based largely on, the child’s personal experience is easier to play, and thus potentially increases perceived realism. In contrast, Brown *et al.* [18] suggest that collaboration with children/adolescents in role creation is inappropriate given the personal and potentially painful nature of past experience. Training young children for consistent role portrayal could be problematic, so distancing the role from their own personal experience may not necessarily be a protective mechanism, rather one that leads to an increased need for training. Closely aligning the role to their developmental stage and personality, or enhancing engagement through inclusion of personal belongings, could perhaps be the ideal method for accurate, consistent and realistic role depiction. Level of SP engagement is critical, but it is not feasible to involve them every time in scenario design.

#### Participation and support

CASPs’ actual participation in the simulation was difficult to ascertain. Only two studies identified the duration of active participation as 90 minutes and an average of 10.1 60–70 minute interviews [17, 23]. An additional 3 studies [27, 29, 30] indicated that CASPs were involved in 4 to 8 consultations per day. Regardless of the actual active participation time, it is clear that in some instances it is ethically inappropriate to repeatedly expose children, especially younger children, to repeated examinations.

A variety of support measures were implemented prior to, during and after the simulation. Austin *et al.* [17] implemented a number of these during the preparation and active phase, including parental presence and nursing support for younger children. A number of studies suggested that the presence of an adult SP is an effective support mechanism [22, 23, 27, 28]. Particularly in risk-taking scenarios, Blake *et al.* [23] identified that developing a relationship with the SP mother can be protective and enabling, thus mitigating the negative impact of involvement. Involving mental health or child communication specialists pre and post simulation was also particularly critical for young children, risk-taking or psychologically stressful roles [25]. In two studies [17, 18], children were also given a method by which they could indicate their desire to end scenario participation. Several studies also recognised the critical need for follow up using either independent interview or focus group methods [18, 20, 21, 23, 24, 25, 27].

Despite the successful involvement of children as young as 6 years of age in simulation (Austin *et al.* [17]), the majority of studies focused on adolescents aged between 11–19 years. This could be attributed to the focus and content of the simulation; however, Tsai [12] suggests that young children are not reliably able to reproduce a role with enough credibility to create realism or consistency.

#### Ethical Issues relating to children as SPs

Given the legal age and developmental stage of children and adolescents, their engagement in SP work raises ethical concerns. Gaining consent from children, adolescents and/or parents is one critical ethical issue. The participation of young children was consented to by parents, although one study (12) does suggest that as young children are unable to understand their role or effects of involvement, consent should not be given particularly where there is no observable benefit for the child. In the absence of benefit, the impetus for safeguarding child participants rests in negating harm. Multiple studies raised the notion that adolescents 16 years or above need not gain parental consent prior to involvement as their cognitive level suggests ability to comprehend the requirements and potential adverse consequences of involvement. However, in most cases, information was provided to adolescents should they wish to inform their parents.

The principles of autonomy, beneficence, and non-maleficence are critical ethical considerations when employing vulnerable populations such as children or adolescents. Respecting the autonomy of children is somewhat difficult, given their developmental inability to make decisions based on informed choices. It presumably then falls to the parents of younger children to determine whether participation as an SP truly reflects the child’s best interests. In the situation where a young child is to be engaged in SP work, the principle of beneficence emerges. Health professionals must make a critical decision regarding their involvement, balancing benefits to the child with the potential for risk or harm to either the child or the learner.

The principle of non-maleficence dictates that harm should be limited and importantly not disproportionate to the benefits of involvement [32]. Younger children are more at risk of adverse outcomes related to under-developed psychological and psychosocial defence mechanisms. ASPs involved in risk-taking, sexuality or mental health scenarios acknowledge a transient negative or discomfort reaction, however there is no evidence to support the presence of long-term adverse effects. The addition of appropriate selection, training, support and debriefing strategies can also serve to ameliorate any deleterious effects (18, 24, 25, 26, 30]. In the decision making process, the risk/harm to benefit ratio must be carefully balanced to ensure the possibility of harm does not outweigh the benefits of involvement.

The potential for harm can be mitigated by reducing the number of examinations, duration of involvement and regular substitution of children to avoid long periods of involvement. There is need to ensure appropriate safeguards are in place prior to, during and post simulation including identification of an ‘exit’ clause for children, whereby if distressed, unsure or anxious, a child can remove themselves from the scenario. Debriefing, including developmentally aware and content specialists, is clearly supportive whilst adequate follow up is also necessary for monitoring potential medium and long-term consequences.

Because ASPs felt they could be could be viewed as risk-takers outside of the research context, coming ‘out of character’ to give feedback was implemented as a psychological safeguard. One study [27] identified that as a result of the vulnerability of adolescents, there is potential they adopt risk-taking behaviours. Whilst this is possible, adolescents in another study reported that the enactment of a substance abuse role actually had a preventive rather than incentive effect [24].

#### The impact of CASP involvement on the learner

Overwhelmingly, the literature suggests that key areas of professionalism, such as communication, are well suited to CASP based simulation. CASP inclusive education and assessment can provide experiential learning opportunities capable of impacting on the preparation of health professionals for clinical work. However, whilst the involvement of CASPs can be beneficial, there is limited evidence that it is actually the child or adolescent who is responsible for positive learning outcomes. In some circumstances, the performance of participants in OSCE who were prepared with an educational program involving ASP simulation surpassed that of others educated using an alternative teaching method. It is difficult to accurately confirm that it was the adolescent, rather than the entire preparatory program, that resulted in better outcomes.

Six studies addressed participant involvement and evaluation as their primary focus, with the majority indicating the positive impact of education programs involving CASP. Austin *et al.* [17] identify the positive impact on learner knowledge and confidence, whilst multiple other studies (18, 20, 23, 28, 30] indicate beneficial aspects of CASP involvement including the achievement of realism and the addition of high level challenge. Despite the myriad of beneficial outcomes, the most powerful one appears to be adolescent feedback. Regardless of whether feedback was given to participants in their role playing persona, or as themselves, adolescent evaluation of the learner’s performance was incredibly powerful [22]. Blake *et al.* [22] in their research involving simulation based education and subsequent OSCE based assessment, further emphasize the powerful nature of feedback indicating that performance improved in an OSCE if the participant received feedback after simulation.

There is distinct variability within the studies regarding outcomes for the learner. Blake *et al.* [22] indicate that interviewing skills can be retained for up to one year if adolescents are involved, whilst in contrast Pullon *et al.* [27] suggest that although a positive experience, education programs involving CASP has little, if any, direct impact on learner clinical performance. The question therefore remains as to whether it is the program alone, or the involvement of children and/or adolescents that results in positive learner outcomes.

Children and adolescents are included in simulation for different purposes including: application and expansion of knowledge, repetitive practice and assessment. This variability in purpose could actually be the factor that impacts on learning to a far greater extent than that which could be attributed solely to CASP involvement. The implementation and management of CASP based programs can be challenging on many levels. There is a fundamental need therefore to carefully consider if their involvement is the critical element of student learning, or the same outcomes would have been achieved with an alternative modality [12].

#### Parents’ perspectives

Parents across all studies identified positive outcomes for children, with the most common responses categorised as the development of knowledge and empowerment, particularly in regards to the preventative nature of risk-taking scenarios, and the opportunity for financial gain. Blake *et al.* [23] identify expanding knowledge in regard to empowerment as a consumer, along with an increased understanding of difficulties associated with being a doctor and importantly, no elevated interest in risk-taking behaviours. Parents also noted positive effects in relation to self-confidence, job skills and sense of responsibility. Lindsey-Lane *et al.* [20] conducted parental focus groups that identified positive outcomes including development of knowledge related to interpersonal dynamics. Parents in this study felt that their child’s participation was a privilege and they were proud of ‘having a real job and earning money’.

Although primarily positive, some parents suggested that SP work was not suitable for all adolescents; rather they emphasized the need for CASP to be self-aware and understand boundaries [23]. In addition, the propensity for training to be scheduled at night, rather than during school hours, impacted on further participation for the adolescent of one parent [24]. Parental feedback garnered by Lindsey-Lane *et al.* [20] indicated that at times children found their involvement boring and tiring. In addition, the need to accompany their child to simulation had financial implications in relation to missing paid employment, travel costs and costs of alternate care provision for other children [12].

#### Child perspectives

The experience for children and adolescents involved in SP work can be positive or negative. Positive impacts include development of knowledge, contributing to the education of future health professionals and financial gain. For adolescents particularly, the preventative nature of risk-taking scenarios is also emphasized as a positive outcome.

Blake *et al.* [23] identified that gaining medical knowledge for adolescents was interesting. However and perhaps more importantly, the ASPs developed empathy for peers with medical problems. The emergence of assertiveness when interacting with their own GP and an elevated understanding of the difference between ‘good and bad doctors’ also proved beneficial. Satisfaction in making an important contribution to the training of future health professionals, having fun, making new friends, and gaining important skills for future employment were considered positive outcomes for CASP [24] as were helping adults learn and knowing those adults valued their input into medical training programs [21]. Younger children particularly felt they were having fun by play-acting, and that their involvement was a good excuse to miss school [21]. Financial gain was repeatedly recognized as a beneficial outcome of involvement [18, 20, 21, 23, 24, 30]. Although in direct contrast, adolescents in one study revealed that money was not a major motivator for participation [24].

Two studies found that enactment of a substance abuse or risk-taking role actually had a preventative, rather than encouraging, effect on adolescents [23, 24]. Involvement in high-risk and mental health simulations also had limited negative effects, with only transient rather than long-term depressive reactions experienced.

Adolescents identified that giving feedback was troublesome, and at times anxiety provoking (18, 30]. Adolescents expressed worry that exhibiting risk-taking behaviour within the scenario would follow them to an external context, and they clearly expressed a desire for feedback to be given in their real persona, rather than in ‘role’ [23]. Losing its glamour and becoming a real job that required commitment was cited as negative (Blake *et al.,* [23]) whilst the impact on social plans because of travel and training schedules was problematic. Missing school and declining school performance, anxiety and tiredness were also identified as significant issues, particularly for younger children [17, 20, 24].

Some adolescents expressed discomfort with the content of some roles. Hanson *et al.* [24] found that sexually explicit questions caused some discomfort for participants who often reacted with anxiety and shock. Roles could also be seen as increasing the adolescents’ worries about their own health or mortality, particularly when they overheard statements about the possible death [21, 24].

## Discussion

This systematic review analysed the literature related to children and adolescents who work as SPs in health professional education. It has indicated that the inclusion of CASPs in education and assessment programs is a viable option for health professions. Fifteen studies arising from various sources and involving different health professions, developmental age groups and focus clearly suggest that CASP involvement is feasible as both a learning and assessment strategy. The review demonstrates that children of various age groups can be involved in simulated case scenarios, short objective structured clinical examinations (OSCE) and clinical skills assessment (CSA).

Several studies did indicate that the content of scenarios should be based on real experience [12, 30]. In addition, research does suggest that matching the developmental age and personality of the child to the required role is also a means to improve performance [12, 21]. This is particularly important for younger children who can find it difficult to portray an actual patient well enough to convince of realism. Overall, studies indicated that even risk taking roles are appropriate if sufficient support is available. However, the literature does clearly inform that scenarios involving death are inappropriate, particularly for younger children [17, 21].

In comparing studies where SPs provided learner feedback, the literature agreed that it results in powerful learning outcomes for participants [18, 23, 24, 30]. Children may not be able to complete long feedback reports, however their ability to deliver concrete and direct feedback is equally as powerful as the adolescent whose feedback tends to be more abstract and reflective [18, 27]. Although children and adolescents may find the provision of feedback difficult [30], the addition of an SP mother to the dyad can guide the process [23].

Outcomes for SPs must be positive if their inclusion in health professional simulation is viable. Interestingly, the power of financial gain is not paramount for SPs. What is of importance is that SPs are able to identify more with their own medical care, and make changes if necessary. SPs became more assertive when assessing the quality of their own medical care, and declared an increased ability to discriminate between good and bad doctors [18, 21, 23, 24]. Even young children, who perhaps are less able to clearly articulate their thoughts regarding medical care, exhibited a strong reaction to poor interpersonal communication [20].

Dissent within the literature is apparent around key areas including: preparation of children and adolescents for the SP role, ethical considerations when employing children, and the impact of the child or adolescent on tangible participant learning outcomes. Inability to realistically portray a role consistently for long periods can affect fairness and reliability of assessment whilst it may also be inappropriate for a child to simulate particular conditions or consent to interventions where there is no benefit. Much of the literature does identify strategies that can be employed to address possible harm Careful selection, preparation, support and debriefing are critical, whilst the inclusion of an SP parent is an addition deemed both supportive and encouraging.

Children and adolescents should only be involved in simulation where benefit clearly outweighs any possible negative outcomes. Where children can either provide assent or consent, there should be clear and developmentally appropriate explanation of the role. In situations where younger children are involved, the literature agrees that parents must be provided with adequate information to enable provision of informed consent prior to their child’s participation.

Although children and adolescents have been involved in simulation for teaching and assessment for many years, they remain under-utilised in health professional education. The reducing nature of clinical placement availability and appropriateness, in conjunction with the patient safety agenda, demands educators adopt a more realistic and feasible strategy to adequately prepare students and professionals for practice.

### Review limitations

Although an extensive search strategy was utilised, the total number of papers included is low. This number may have been reduced due to exclusion of papers written in languages other than English. Although the process and tools utilised for data collection were mostly robust and validated, the inclusion of self-evaluation processes may not be as reliable in capturing learning outcomes. The sample size of studies focusing on child and adolescent SPs rather than the learner was also quite low. Given this, the ability to extrapolate data to different age groups, health professional groups or clinical practice environments could be limited. Despite these limitations, the review suggests that employment of children and adolescents in health professional education is feasible and there are demonstrable positive outcomes for both learners and child/adolescent SPs.

## Conclusion

The findings of this systematic review suggest that simulation based education and assessment programs involving children and adolescents are feasible and capable of producing positive outcomes for both CASPs and participants. There remains inherent variability in recruitment and preparation, developmental stage of CASPs and type of role they portray. The collective studies indicate that CASP involvement in paediatric simulation endeavours can enhance realism and preparation of health professional students for work, although further research is required to isolate the specific benefit of interacting with children and adolescents. The literature clearly suggests that consideration of ethical principles including autonomy, beneficence and non-maleficence, is a critical element of CASP programs. While there is recognition of the potential for negative outcomes, these can be managed.

### Recommendations for future research

There is a lack of research regarding CASP based programs in nursing education despite a clear need for objective analysis of their impact on learning outcomes and assessment results. The significance of this could be seen as contentious given the ability to extrapolate from other health professional domains. However, it would however be fruitful for nursing education to have credible research on which to base and expand nursing specific simulation. The impact on longer term outcomes such as retention of knowledge and skill learning is also critical as the demand to produce simulation capable of exerting an impact, rather than just being enjoyable, grows.

A gender bias is obvious throughout the studies, with the majority of literature focused on adolescent females with the dyad of clinician and mother/daughter presentation also dominant. Expansion of studies to include younger children may be of benefit to future education endeavours, as would the involvement of males. These are particularly important given the potential for both these groups to need health care. Within a multicultural society where cultural and linguistic diversity exists, adequate exposure of the learning group to CASPs and families with English as a second language and varying cultural mores and values would be beneficial.

Although feedback is gained from CASPs, there are multiple studies where their voice in evaluation and assessment is absent. Incorporating developmentally appropriate strategies to enable provision of feedback from all age groups is one option for ensuring a breadth of feedback is received.

If health professional education programs continue to ponder replacing at least some proportion of clinical hours with simulation, the need to ensure experience and learning is equitable with placement outcomes is essential. Incorporating children and adolescents in simulation is one way of fostering this outcome.

## References

[1] Davies J, Nathan M, Clarke D (2012). An evaluation of a complex simulated scenario with final year undergraduate children’s nursing students. Collegian.

[2] Hayden JK, Smiley RA, Alexander M, Kardong-Edgren S, Jeffries P (2014). The NCSBN National Simulation Study: A Longitudinal, Randomized, Controlled Study Replacing Clinical Hours with Simulation in Prelicensure Nursing Education. Journal of Nursing Regulation.

[3] Jones DC, Sheridan ME (1999). A case study approach: Developing critical thinking skills in novice pediatric nurses. The Journal of Continuing Education in Nursing.

[4] Hovancsek M, Jeffries PR (2007). Using simulation in nurse education. Simulation in Nursing Education: From conceptualization to evaluation.

[5] Wind LA, Dalen JV, Muijtjens AM, Rethans J (2004). Assessing simulated patients in an educational setting: the MaSP (Maastricht Assessment of Simulated Patients). Med Educ.

[6] Barrows HS (1968). Simulated patients in medical teaching. Can Med Assoc J.

[7] Bornais JAK, Raiger JE, Krahn RE, El-Masri MM (2012). Evaluating Undergraduate Nursing Students’ Learning Using Standardized Patients. J Prof Nurs.

[8] Ker JS, Dowie A, Dowell J, Dewar G, Dent JA, Ramsay J (2005). Twelve tips for developing and maintaining a simulated patient bank. Med Teach.

[9] Webster D (2014). Using standardized patients to teach therapeutic communication in psychiatric nursing. Clinical Simulation in Nursing.

[10] Cleland JA, Abe K, Rethans JJ (2009). The use of simulated patients in medical education: AMEE Guide No 42. Med Teach.

[11] Haddington N, Hanning L, Weiss M, Taylor D (2013). The use of a high-fidelity simulation manikin in teaching clinical skills to fourth year undergraduate pharmacy students. Pharm Educ.

[12] Tsai T (2004). Using children as standardised patients for assessing clinical competence in paediatrics. Arch Dis Child.

[13] Child employment laws and requirements. State Government of Victoria, Melbourne. 2015. http://www.business.vic.gov.au/hiring-and-managing-staff/employing-children/laws-and-act. Accessed 16th October 2015.

[14] Liberati A, Altman DG, Tetzlaff J, Mulrow C, Gøtzsche PC, Ioannidis JPA (2009). The PRISMA statement for reporting systematic reviews and meta-analyses of studies that evaluate healthcare interventions: explanation and elaboration. BMJ.

[15] Walsh D, Downe S (2006). Appraising the quality of qualitative research. Midwifery.

[16] Reed DA, Beckman TJ, Wright SM, Levine RB, Kern DE, Cook DA (2008). Predictive validity evidence for medical education research study quality instrument scores: Quality of submissions to JGIMs medical education special issues. JGIM: Journal of General Internal Medicine.

[17] Austin EN, Hannafin NM, Nelson H (2013). Pediatric disaster simulation in graduate and undergraduate nursing education. J Pediatr Nurs.

[18] Brown R, Doonan S, Shellenberger S (2005). Using children as simulated patients in communication training for residents and medical students: A pilot program. Acad Med.

[19] Feddock CA, Hoellein AR, Griffith CH, Wilson JF, Lineberry MJ, Haist SA (2009). Enhancing knowledge and skills through an adolescent medicine workshop. Archives Of Pediatrics & Adolescent Medicine.

[20] Lindsey-Lane J, Ziv A, Boulet J (1999). A pediatric clinical skills assessment using children as standardized patients. Archives of Pediatric Adolescent Medicine.

[21] Woodward CA, Gliva-McConvey G (1995). Children as standardized patients: Initial assessment of effects. Teach Learn Med.

[22] Blake K, Mann KV, Kaufman DM, Kappelman M (2000). Learning adolescent psychosocial interviewing using simulated patients. Acad Med.

[23] Blake KD, Gusella J, Greaven S, Wakefield S (2006). The risks and benefits of being a young female adolescent standardised patient. Med Educ.

[24] Hanson M, Tiberius R, Hodges B, Mackay S, McNaughton N, Dickens S (2002). Adolescent standardized patients: Method of selection and assessment of benefits and risks. Teaching and Learning in Medicine: An International journal.

[25] Hanson M, Niec A, Pietrantonio AM, Johnson S, Young M, High B (2007). Effects Associated with Adolescent Standardized Patient Simulation of Depression and Suicidal Ideation. Acad Med.

[26] Hanson M, Niec A, Pietrantonio AM, Johnson S, Young M, High B (2008). Does Mental Illness Stigma Contribute to Adolescent Standardized Patients’ Discomfort With Simulations of Mental Illness and Adverse Psychosocial Experiences?. Acad Psychiatry.

[27] Pullon S, McKinlay E, Wynn-Thomas S (2003). Teaching complex consultation skills using adolescent and parent simulated patients. Focus on Health Professional Education: A Multi-disciplinary journal.

[28] Rowe AK, Onikpo F, Lama M, Deming MS (2012). Evaluating health worker performance in Benin using the simulated client method with real children. Implement Sci.

[29] Bokken L, Van Dalen J, Scherpbier A, Van Der Vleuten C, Rethans J (2009). Lessons learned from an adolescent simulated patient educational program: five years of experience. Med Teach.

[30] Bokken L, Van Dalen J, Rethans J (2010). The case of “Miss Jacobs”: Adolescent simulated patients and the quality of their role playing, feedback, and personal impact. Simul Healthc.

[31] Braun V, Clarke V (2006). Using thematic analysis in psychology. Qual Res Psychol.

[32] Beauchamp TL, Childress JF (2009). Principles of Biomedical Ethics.

